# Draft Genome Sequence of Cyanobacterium *Hassallia byssoidea* Strain VB512170, Isolated from Monuments in India

**DOI:** 10.1128/genomeA.00064-15

**Published:** 2015-03-05

**Authors:** Deeksha Singh, Mathu Malar Chandrababunaidu, Arijit Panda, Diya Sen, Sourav Bhattacharyya, Siba Prasad Adhikary, Sucheta Tripathy

**Affiliations:** a Structural Biology and Bioinformatics Division, CSIR, Indian Institute of Chemical Biology, Kolkata, West Bengal, India; b Fakir Mohan University, Vyasa Vihar, Nuapadhi, Balasore, Odisha, India

## Abstract

The draft genome assembly of Hassallia byssoidea strain VB512170 with a genome size of ~13 Mb and 10,183 protein-coding genes in 62 scaffolds is reported here for the first time. This is a terrestrial hydrophobic cyanobacterium isolated from monuments in India. We report several copies of luciferase and antibiotic genes in this organism.

## GENOME ANNOUNCEMENT

Cyanobacteria are phototrophs that can survive in different environmental conditions. The photosystem I and II genes evolved in different lineages of bacteria and were acquired by cyanobacteria through lateral gene transfer (1). Cyanobacteria have been well known for the production of various bioactive compounds (2). Increasing environmental pollution from industrial effluents containing hazardous chemicals is currently a severe concern. Many strains of cyanobacteria can be efficiently used in bioremediation (3), including *Hassalia* sp., which was shown to degrade synthetic polymers (4). Hassallidins, a group of glycosylated lipoproteins having antifungal properties, are end products of several nonribosomal protein synthetic pathways produced by *Hassallia* sp. (5).

*Hassallia byssoidea* VB512170 was isolated as a greenish-brown terrestrial organism from stone monuments of India (6). H. byssoidea VB512170 cultures were grown in BG11 medium under 16-h light and 8-h dark conditions at ~28°C without shaking. It is a filamentous, heterocystous, hydrophobic strain that grows well on solid media and floats on the surface of liquid media. Morphologically, these cultures produce stabbing-like depressions on the surface of solid media.

Genomic DNA was extracted and purified using a Uniflex Bacterial DNA isolation kit (Genei, USA). One microgram of DNA was provided for shotgun sequencing and 3 µg for mate-pair sequencing. Genome sequencing was carried out on the Illumina HiSeq platform. Paired-end libraries having an insert size of 300 bp and read length of 151 bp at 94× coverage generated approximately 10.2 million reads. Mate-pair libraries having an insert size of 3,000 bp and read length of 101 bp at 59.7× coverage generated 5 million reads. We used a variety of approaches for assembly. In the first phase, we used the A5 pipeline (7) for read cleaning and genome assembly. The contigs produced from this assembly were randomly sheared into 6-kb and 20-kb paired-end jump libraries at 10X coverage with a read length of 101 bp using the wgsim tool (8). In the second phase, we used Allpaths-LG-49856 (9) on 3-kb paired-end and 6-kb and 20-kb mate-pair libraries for final assembly. The final assembly with a genome size of 13 Mb (13,096,531 bases), an *N*_50_ value of 1,254,578 bp, and a total G+C content of 44% was generated. The assembly had 62 scaffolds from 257 contigs; the largest scaffold was 1,860,517 bp long, while the smallest was 2,000 bp long.

Genome annotation of the scaffolds was performed using PGAP (http://www.ncbi.nlm.nih.gov/genome/annotation_prok). A total of 10,183 protein-coding genes, 1,322 pseudogenes, 7 CRISPR genes, 18 rRNA genes, 135 tRNA genes, and 2 noncoding RNA genes were predicted from this genome. The genome carries few copies of the luciferase genes and genes coding for mercury and arsenic reductases (*arsC* and *arsB*), which can be extremely useful from a bioremediation point of view. BLASTp of mercuric reductase against the NR database showed 99% identity with Scytonema hofmanni UTEX 2349. The arsenic resistance protein ArsB showed 89% identity with its homolog on Crinalium epipsammum.

### Nucleotide sequence accession number.

The *Hassallia byssoidea* VB512170 genome sequence and annotation data have been deposited in GenBank under the accession number JTCM00000000.
